# Bone Mesenchymal Stem Cell-Derived Extracellular Vesicles Containing Long Noncoding RNA NEAT1 Relieve Osteoarthritis

**DOI:** 10.1155/2022/5517648

**Published:** 2022-04-15

**Authors:** Shuai Zhang, Zhe Jin

**Affiliations:** Department of Orthopaedics, The First Hospital of China Medical University, Shenyang 110001, China

## Abstract

Extracellular vesicles (EVs) derived from bone marrow mesenchymal stem cells (BMSCs) possess potentials in modulation of the biological process in various diseases. However, an extensive investigation of the mechanism of BMSC-derived EVs (BMSC-EVs) in osteoarthritis (OA) remains unknown. Thus, we focused on the mechanism behind BMSC-EVs in OA. Cartilage tissues were harvested from OA patients, in which the microRNA (miR)-122-5p and Sesn2 expression were determined. BMSCs and their EVs were extracted. Chondrocytes were cocultured with BMSC-EVs overexpressing NEAT1, followed by gain- or loss-of-function assays for studying their effect on cell proliferation, apoptosis, and autophagy. Relationship among NEAT1, miR-122-5p, and Sesn2 was assessed. OA mouse model was established by the destabilization of medial meniscus method to elucidate the effect of NEAT1 in vivo. NEAT1 could be transferred from BMSC-EVs into the chondrocytes. miR-122-5p was highly expressed but Sesn2 was poorly expressed in cartilage tissues of OA patients. Mechanically, NEAT1 bound to miR-122-5p to limit miR-122-5p expression which targeted Sesn2, thus activating the Nrf2 pathway. In chondrocytes, NEAT1 delivered by BMSC-EVs, miR-122-5p downregulation, or Sesn2 overexpression induced the proliferation and autophagy of chondrocytes but inhibited their apoptosis. Meanwhile, NEAT1 delivered by BMSC-EVs relieved OA by regulating the miR-122-5p/Sesn2/Nrf2 axis in vivo. Taken altogether, BMSC-EVs containing NEAT1 activated the Sesn2/Nrf2 axis via binding to miR-122-5p for protection against OA.

## 1. Introduction

Osteoarthritis (OA) has been regarded as a vital cause of disability and economic burden among senior citizens, and a significant health issue for many over the age of 65 [1–3]. OA is the most prevalent chronic joint disease in the world, both incidence and prevalence of which are expected to rise with the increasing age [4]. In addition to the old age, obesity, female gender, knee injury, overuse of joints, bone density, muscle weakness, and joint laxity all are predisposing causes for the development of OA [5]. Treatment approaches for this disease constitute the reduction of modifiable risk factors, and intraarticular, physical, alternative, and surgical therapies [6]. Despite the advancements made in the treatment protocols for OA, the quality of patients' life after prognosis is still moderate, and detecting in early-stage lacks sensitivity and warrants for superior sensitive outcomes than the current methods [2]. The progression of OA entails the involvement of chondrocyte proliferation and viability and novel therapies targeting chondrocyte alterations like cell apoptosis, proliferation, and autophagy show great potentials [7]. In light of the aforementioned literature, a deeper insight is warranted into the mechanism of chondrocyte changes underlying OA to make targeted therapy to present its therapeutic potential.

Bone marrow mesenchymal stem cells (BMSCs) are multipotent cells derived from bone marrow, which possess the abilities to differentiate into osteoblasts, adipogenic cells, and chondroblasts [8]. BMSCs are indicated in cartilage tissue engineering in an attempt to treat cartilage lesions or OA [9]. Notably, BMSCs may affect skeletal diseases by secretion of extracellular vesicles (EVs) [10]. EVs, membranous vesicles with diameter of 40-150 nm, have been identified as transduction molecules for extracellular signal, serving as delivery agents of long noncoding RNAs (lncRNAs), microRNAs (miRNAs or miRs), proteins, and lipids [11]. lncRNAs, a type of noncoding RNA that comprises of longer than 200 nucleotides sequences, participate in cellular events such as proliferation, migration, differentiation, and apoptosis, and their dysregulation is involved in different types of cancer [12, 13]. miRNAs are short noncoding RNAs possessing the capacity of modulating target gene expression at posttranscriptional level, which share correlation with cancer development due to their effect on vital biological mechanisms including apoptosis, migration, and differentiation [14, 15]. lncRNAs can serve as a competing endogenous RNA (ceRNA) for miRNAs and thereby reduce the expression of miRNAs [16, 17]. An existing study demonstrated the enrichment of lncRNA nuclear paraspeckle assembly transcript 1 (lncRNA NEAT1) in EVs [18]. Correspondingly, the involvement of NEAT1 in the mediation of OA has been identified in association with miR-181c by mediating synoviocytes [19]. Notably, the ability of NEAT1 to negatively regulate the miR-122 expression to affect liver fibrosis has been documented [20]. miR-122-5p is one of the circulating miRNAs involved in the pathogenesis of OA [21]. However, the NEAT1/miR-122-5p axis is rarely studied in OA. Additionally, Sestrins (Sesn) is aberrantly expressed in OA with an undefined mechanism [22], which was worthy of further exploration. Here, we sought to explore the pathological mechanism of OA and the interaction among NEAT1, miR-122-5p, and Sesn in OA using mouse model, in an attempt to identify an appealing therapeutic modality for OA treatment.

## 2. Methods

### 2.1. Ethics Statement

The clinical study was implemented under the ratification of the Ethics Committee of the First Hospital of China Medical University and the guidance of the *Declaration of Helsinki*. Informed consent form was filled in by all subjects prior to participation. Animal experiments were implemented under the ratification of the Animal Ethics Committee of the First Hospital of China Medical University. We tried our best to limit animals' suffering.

### 2.2. Clinical Samples

OA cartilage tissues were isolated from patients with OA (*n* = 25; aged 59-65 years) who underwent total knee arthroplasty at the First Hospital of China Medical University. Normal cartilage tissues were isolated from patients with acute injury or amputation (*n* = 25; aged 42-47 years). No patients received any treatment before biopsy. Human BMSCs were isolated from three cases of donated bone marrow of OA patients with reference to the established protocols [23]. A conclusive diagnosis was confirmed utilizing magnetic resonance imaging (MRI). Patients without femoral-head height loss were selected, and those with systemic diseases, including trauma, infiltration of blood system, binding, and tumors, were excluded.

### 2.3. Culture of Primary Chondrocytes

Under sterile conditions, the cartilage tissues of the patients were washed repeatedly with phosphate buffer saline (PBS) to remove blood stains, after which the attached synovium and fibrous tissues were removed and divided into sections of 1 mm^3^ in size. The sections were digested with 0.05% collagenase type II (Sigma-Aldrich, St. Louis, MO) for 30 min and centrifuged at 1,000 r/min. Next, 0.1% collagenase type II and 0.25% trypsin (Sigma-Aldrich) were added into the section at 37°C for 60-100 min. Dulbecco's modified Eagle's medium (DMEM; Gibco, Grand, NY) appended to 10% FBS, 100 U/mL penicillin, and 100 U/mL streptomycin was then added for dissolution of the cell mass. The cells were seeded in culture flasks (4 × 10^5^ cells/mL) and cultured at 37°C and 5% CO_2_. The cells were passaged for subsequent experiments.

### 2.4. Cell Transfection

Lentivirus overexpressing NEAT1 (Lv-NEAT1), or carrying negative control (Lv-NC), sh-NEAT1, sh-Sesn2, or sh-NC were packaged into HEK-293T cells (ATCC, Manassas, VA; cultured in DMEM appended to 10% FBS and 1% penicillin-streptomycin at 37°C and 5% CO_2_) with a lentiviral packaging kit (Invitrogen, Carlsbad, CA). Viral supernatants were collected after 48 h, and the viral concentration was adjusted by GeneChem (Shanghai, China). Upon achieving approximately 50% confluence, BMSCs were transduced with lentivirus and screened with 10 *μ*g/mL puromycin (Sigma-Aldrich) 48 h after transduction. After screening of the stably transfected cell lines, multiple shRNAs were designed, and the sequence with the highest interference efficiency was chosen for subsequent experimentations.

miR-122-5p mimic/inhibitor, si-Sesn2, si-NEAT1, and corresponding NCs were brought from GeneChem, and chondrocytes were cultured in a 6-well plate (4 × 10^5^ cells/mL). Cell transfection was implemented utilizing Lipofectamine 2000 reagent (11668-019, Invitrogen) under 80% confluence. After culture for 48 h at 37°C and 5% CO_2_, the cells were gained for subsequent experimentations.

### 2.5. Identification of BMSCs

The isolated BMSCs were cultured in DMEM-F12 basal medium (Hyclone, Germany) appended to 10% FBS (10099141, GIBCO), 0.2% penicillin and streptomycin (Hyclone), and passaged once every 3 days. The subsequent experiments were implemented utilizing BMSCs at passage 3-7.

### 2.6. Flow Cytometry for Identification of BMSCs

The BMSCs at passage 3 with 80% confluence were chosen here. Following removal of culture medium, the cells were subjected to digestion and centrifugation with the precipitate collected. After washing twice with PBS, cell counting was performed. The cells (1 × 10^6^ cells/mL) were transferred in a 15 mL centrifuge tube with 100 *μ*L PBS buffer appended to 2% FBS. Specific fluorescent flow antibodies against CD73, CD90, CD105, and CD44 labeled by PE, or CD34, CD45, and CD14 labeled by FITC (1: 100, all from BD Biosciences, San Jose, CA, rat antimouse), were added into cells for incubation at 4°C for 30 min (dark condition). Then, the cells were resuspended in 3 mL PBS, centrifuged, and reacted with 300 *μ*L PBS buffer. The background markers were determined by the presence of the homotypic monoclonal antibody in the control group. The fluorescent cells were checked utilizing a flow cytometer (BD FACSVerse, CA). The positive rate of surface antigen was measured with the help of FlowJo (FlowJo, LLC, Ashland, OR), which was presented as %.

### 2.7. Isolation of EVs from BMSCs (BMSCs-EVs)

FBS was precentrifuged at 1,000 r/min for 18 h to eliminate EVs from serum. Under 80% confluence, the supernatant from the culture medium was removed, with replacement of the 10% EV-depleted FBS culture solution. Next, the cells were further cultured in a CO_2_ incubator at 37°C for 48 h. The collected supernatant was centrifuged at 500 g for 15 min, with removal of the cell debris at 4°C. Subsequently, the cells were centrifuged at 2,000 g for 15 min, with removal of the cellular debris or apoptotic bodies at 4°C. Large vesicles were removed by a 20 min regimen of centrifugation at 1,000 r/min and 4°C. The cells were centrifuged for 70 min at 1,000 r/min after 0.22 *μ*M filter filtration, resuspended in PBS at 4°C and then with 100 *μ*L of sterile PBS. All ultracentrifugations were performed with SW-32Ti rotors using the Beckman ultracentrifuge (Optima L-90 K) at 4°C. The remaining low-speed centrifugations were conducted using the Beckman Allegra X-15R centrifuges.

### 2.8. Identification of EVs

The particle size distribution of the EVs was checked by nanoparticle tracking analysis (NTA) (Malvern Instruments Ltd., UK).

For transmission electron microscope (TEM), 20 *μ*L of the isolated EVs was loaded on a carbon-coated copper electron microscope grid for 2 min and labeled with the phosphotungstic acid solution (12501-23-4, Sigma-Aldrich) for 5 min. The grids were then kept semidry with filter paper. Images were captured at 80 kV under a Hitachi H7650 TEM (Japan).

The expression of EV-specific markers TSG101 (ab30871, Abcam, Cambridge, UK), CD81 (ab79559, Abcam), and Alix (ab76608, Abcam) was detected utilizing Western blot analysis.

### 2.9. Uptake by EVs by Chondrocytes

Purified BMSCs-EVs were labeled with PKH26 red fluorescence kit (Sigma-Aldrich). Next, the EVs were resuspended in 1 mL diluent C solution, and 4 *μ*L of PKH26 ethanol staining solution was added to prepare a 4 × 10^−6^ M dye solution. A total of 1 mL of the EV suspension was mixed with dye solution for 5 min, and 2 mL of 1% EV-free FBS added for incubation for 1 min to terminate staining. The labeled EVs were ultracentrifuged at 1,000 r/min for 2 h, and the EVs in the samples were enriched in sucrose at a density of 1.13-1.19 g/mL, followed by the collection of the EVs. PKH26-labeled EVs were cocultured with chondrocytes at 37°C for 12 h. Next, the cells were fixed with 4% paraformaldehyde, and the nuclei were stained with 4′,6-diamino-2-phenylindole (DAPI, D9542, Sigma-Aldrich). Finally, the uptake of EVs by chondrocytes was observed under a fluorescence microscope (ECLIPSE E800, Nikon, Tokyo, Japan).

To explore the transfer of NEAT1, FITC-NEAT1 (green) was transfected into BMSCs utilizing Lipofectamine 2000 reagent, and BMSC-EVs were then collected as the previous method and added with Dil tag (red). Afterward, the BMSC-EVs were cocultured with chondrocytes for 48 h, and the uptake of BMSC-EVs carrying FITC-NEAT1 by chondrocytes was observed under a fluorescence microscope (ECLIPSE E800, Nikon, Japan).

### 2.10. CCK-8 Assay

Chondrocytes were seeded in 96-well plates (5 × 10^3^ cells/well). CCK-8 solution (10 *μ*L, Dojindo, Japan) was supplemented to each well for incubation for 1-4 h at 37°C. A microplate reader (Thermo Fisher Scientific) was utilized for testing the absorbance value at the excitation wavelength of 450 nm.

### 2.11. Flow Cytometry for Detection of Cell Apoptosis

Chondrocytes were rinsed, after which the degree of cell apoptosis was evaluated with the Annexin V-FITC/PI apoptosis detection kit (BD Pharmingen, Franklin Lake, NJ). A flow cytometer (BD Biosciences) was employed for subsequent detection.

### 2.12. Isolation of Mitochondria

The cartilage tissues were rapidly fragmented in ice-cold MSE, added to the buffer, and ground in MSE buffer using a polytron tissue grinder, followed by two rapid grinding regimens with a loose Potter Elvenhjem tissue grinder. The supernatant was preserved after centrifugation of the homogenate. The granular mitochondria were extracted twice from the supernatant and the granules were rinsed with MSE buffer. The final particles were rinsed and resuspended in the medium. Mitochondria were incubated on ice for 15 min, and the protein concentration was evaluated with the help of the Bradford method employing bovine serum albumin (BSA) as standard. All procedures were conducted on ice.

### 2.13. Monodansylcadaverine (MDC) Staining

MDC staining kit (C3018S, Beyotime) was used for this assay. Chondrocytes were placed on a cover glass in a petri dish, which was added with medium. When the cells grew to an appropriate density, the culture medium was removed. The cells were incubated with an appropriate amount of working solution containing MDC probes prewarmed at 37°C (working solution: the staining solution was diluted to the working concentration with 1× Wash Buffer: MDC staining volume ratio = 9 : 1) for 1 h. The above dye solution was washed with PBS, and the cells were observed under a fluorescence microscope. Cells were examined under a fluorescence microscope (with maximum excitation wavelength at 335 nm and maximum emission wavelength at 518 nm). The relative fluorescence intensity was analyzed by analysis software. Three regions of interest were selected for each group on 3 separate photographs. The NC was set at 0 n, which was adjusted as the standard to exclude nuclear fluorescence.

### 2.14. Autophagy Formation Traced by mRFP-GFP-LC3 Double Fluorescence Assay

Chondrocytes were cultured on coverslips for 24 h and then transfected with pmCherry-GFP-LC3 plasmid utilizing Lipofectamine 2000 reagent (Invitrogen) for 24 h. Next, the cells were fixed with 4% paraformaldehyde and observed with a confocal fluorescence microscope (Olympus FV10i, Japan). The number of GFP and mRFP dots was checked according to previously described protocols [24].

### 2.15. RNA Isolation and Quantification

Total RNA from tissues and cells was extracted utilizing TRIzol reagent (15596026, Invitrogen), and then reversely transcribed into cDNA with the help of Prime Script RT Master Mix kit (Takara, RR037B, Tokyo, Japan). RT-qPCR was implemented applying the SYBR Premix Ex Taq II kit (Takara, Cat No.2) with an equal amount of complementary DNA (cDNA) in the 20 *μ*L reaction mixture. The TaqMan MicroRNA Reverse Transcription Kit (Thermo Fisher Scientific; 4366596) was adopted for reverse transcription and relative quantification of the miRNAs. All primers are shown in Supplementary Table 1, and TaqMan probes were purchased from Thermo Fisher Scientific (Cat no. 1). U6 was adopted as an internal reference of miRs and GAPDH of the remaining genes. With determination of the NEAT1 expression in EVs, an endogenous reference for synthesis of 0.1 ng (1.8 × 10^8^ copies) lambda polyA + RNA (Takara) was added at the beginning of RNA reverse transcription to normalize the total RNA in EVs. The relative transcription level of the target gene was calculated utilizing the 2^-△△CT^ method.

### 2.16. Western Blot Analysis

The total protein of tissues and cells was extracted by high-efficiency RIPA lysis buffer (R0010, Solarbio). After centrifugation at 15,000 rpm/min for 15 min and lysis at 4°C for 15 min, the supernatant was harvested, and the protein concentration was evaluated employing the bicinchoninic acid kit (20201ES76, Yeasen Biotechnology, Shanghai, China). Following separation utilizing polyacrylamide gel electrophoresis, the protein was transferred onto PVDF membranes which were sealed at 5% BSA for 1 h at ambient temperature and probed with the diluted primary antirabbit antibodies (all from Abcam) against Sesn2 (ab178518, 1: 10000), Srx1 (ab203613, 1: 10000), Trx1 (ab273877, 1: 10000), Beclin-1 (ab210498, 1: 10000), LC3A/B (ab128025, 1: 1000), Ki67 (ab92742, 1: 10000), Aggrecan (ab3778, 1: 10000), MMP3 (ab39012, 1: 10000), GRP94 (ab238126, 1: 10000), APOB (ab20737, 1: 10000), and GAPDH (ab8245, 1: 10000) overnight at 4°C. The next day, the membrane was reprobed with HRP-labeled goat antirabbit IgG (ab205718, 1: 20000, Abcam) for 1 h at ambient temperature. The developing solution was added for development. ImageJ 1.48 software (National Institutes of Health) was employed for protein quantitative analysis, and protein quantitative analysis was implemented with the gray value ratio of each protein to the internal reference GAPDH.

### 2.17. Dual-Luciferase Reporter Assay

NEAT1 sequence containing the binding site of miR-122-5p and the mutation binding site was cloned into the pGL3-promoter-luciferase reporter vector (Promega, Madison, WI) for construction of the NEAT1-wild type (WT) and NEAT1 mutant type (MUT) reporter vectors. HEK-293T cells were cotransfected with miR-122-5p mimic or mimic-NC and the above reporter vectors utilizing Lipofectamine 2000 reagent (Invitrogen). After 24 h, the luciferase activity was tested with the help of a dual-luciferase reporter assay system (E1910, Promega). The binding ability of miR-122-5p to Sesn2 was detected in compliance with the preceding procedures. Plasmids of miR-122-5p mimic or mimic-NC were transfected into HEK-293T cells together with luciferase reporter pGL3-Luciferase Reporter vector (Promega) containing WT Sesn2-3′UTR or MUT Sesn2-3′UTR.

NEAT1 cDNA was cloned into mammalian expression vector pcDNA3.1 (Invitrogen). NEAT1 cDNA fragments contained predicted potential miRNA binding sites (WT) or disrupted miRNA binding site sequences (MUT). Pmir-RB-Sesn2 or Pmir-RB-Sesn2 mutants were transfected into human chondrocytes with pcDNA3.1, pcDNA3.1-NEAT1, pcDNA3.1-NEAT1-MUT, pcDNA3.1 − NEAT1 + miR − 122 − 5p mimic, or mimic-NC employing Lipofectamine 2000 reagent, respectively. After 48 h of transfection, the relative luciferase activity was calculated.

### 2.18. RIP Assay

The RIP assay was implemented employing the Magna-RNA binding protein immunoprecipitation kit (Millipore, Billerica, MA). RIP buffer containing magnetic beads conjugated with human anti-Ago2 antibody or normal mouse IgG (NC) were added to the whole cell lysate, which was incubated with proteinase K sample for isolation of the immunoprecipitated RNA. The concentration of RNA was analyzed employing a spectrophotometer (Thermo Fisher Scientific) while RNA quality was assessed by a bioanalyzer (Agilent, Santa Clara, CA). Finally, RNA was extracted, and the purified RNA was tested with the help of PCR to verify the presence of binding targets.

### 2.19. RNA Pull-Down Assay

Chondrocytes were transfected with the biotin-labeled WT NEAT1 and biotin-labeled MUT NEAT1 (50 nM each). After 48 h of transfection, the isolated cells were rinsed with PBS and reacted with specific cell lysate (Ambion, Austin, Texas) for 10 min. Subsequently, 50 mL of the sample cell lysis buffer was subpacked. The lysate residue was reacted with M-280 streptavidin magnetic beads (Sigma-Aldrich) precoated with RNase-free and yeast tRNA (Sigma-Aldrich). RNA was extracted and detected by RT-qPCR.

### 2.20. Fluorescence In Situ Hybridization (FISH)

FISH detection was performed employing a FISH kit (Guangzhou RiboBio Co., Ltd., Guangzhou, Guangdong China). FITC-labeled NEAT1 antisense probe 1 and Cy3-labeled miR-122-5p mimic antisense probe 2 were designed and synthesized by RiboBio. Cells were fixed with 4% formaldehyde for 15 min, permeabilized with PBS appended to 0.5% TritonX-100 for 30 min at 4°C, and prehybridized in prehybridization solution at 37°C for 30 min. The probes were then added to the hybridization solution and incubated with the cells overnight at 37°C (dark condition). The next day, cells were stained with DAPI and photographed under a fluorescence microscope (BXF-200, Shanghai Bingyu Optical Instrument Co., Ltd., Shanghai, China).

### 2.21. Establishment of OA Mouse Models

Adult C57BL/6 male mice (5-6 months old; Beijing Vital River Laboratory Animal Technology Co., Ltd., Beijing, China) were housed individually in the SPF laboratory at 22-25°C and 60-65% humidity (12 h of light and 12 h of dark, eat and drink freely). The mice were subjected to destabilization of the medial meniscus (DMM) to establish a mouse model of OA after one week of acclimation. All mice were randomly divided into five groups (6 mice in each group) as the sham group, the DMM group (OA mice with intra-articular injection of PBS), the DMM + Lv − NC − BMSCs − EVs group (OA mice with intra-articular injection of 10 *μ*g EVs isolated from BMSCs), the DMM + Lv − NEAT1 − BMSCs − EVs group (OA mice with intra-articular injection of EVs from BMSCs transduced with lentivirus carrying NEAT1), and the DMM + Lv − NEAT1 − BMSCs − EVs + sh − Sesn2 group (OA mice with intra-articular injection of EVs from BMSCs transfected with lentivirus carrying NEAT1 and sh-Sesn2). The articular cavity of the mice was injected with 10 *μ*g EVs or an equivalent amount of PBS, twice a week for 1 month. The mice were euthanized after 7 weeks of growth after injection. The contents of MMP13 and Aggrecan in the cartilage tissues were detected by immunohistochemistry. The osteoarthritis research society international (OARSI) scoring system [25] on the bone and joint in mice is shown in Supplementary Table 2.

### 2.22. Histological Analysis

The removed cartilage tissue blocks of mice were fixed in 10% formalin and Bouin's fixative solution, rinsed with running water for 30 min, and dehydrated by different concentrations of alcohol. The tissue blocks were cleared in xylene, embedded in paraffin, and sectioned. The sections were stained after dewaxing.

MMP13 and Aggrecan protein expression and distribution in the cartilage tissues were detected by immunohistochemistry. Paraffin specimens of cartilage tissues of mice in each group were sectioned (thickness of 4 *μ*m), dewaxed, and hydrated. The operation was conducted in strict accordance with the conventional immunohistochemical staining method. The sections were then immunostained with antibodies to MMP13 (1 : 500, sc-515284, Santa Cruz Biotechnology, Inc., Santa Cruz, CA) and Aggrecan (1 : 200, sc-33695, Santa Cruz). A total of 5 lesion areas were randomly selected under a 200-fold microscope for counting the number of positive stained cells in the cells [26].

### 2.23. TUNEL

Tissue sections were dewaxed, rehydrated, and subjected to antigen retrieval using the proteinase K working solution and permeating treatment. The sections were incubated with the mixture of TDT and dUTP at a ratio of 1 : 9 at 37°C for 2 h. Endogenous peroxidase (POD) activity was subsequently terminated, and the tissues were covered with a transformer-POD. The tissue sections were developed with DAB, counterstained with hematoxylin for 3 min, dehydrated with gradient ethanol (70%, 80%, 95%, and 100%), cleared in xylene, and sealed with resin sealant. The nuclei stained with hematoxylin were blue, while the positive cells stained with DAB were brown-yellow. All sections were observed under a microscope and analyzed employing Image-Pro Plus 6.0 software (Media Cybernetics, Rockville, MD).

### 2.24. Statistical Analysis

SPSS 21.0 statistical software (IBM Corp., Armonk, NY) was adopted for statistical analysis of data. Measurement data were described as mean ± standard deviation and compared utilizing independent sample *t* test (two groups), one-way analysis of variance (ANOVA) followed by Tukey's post hoc test (multiple groups), or repeated-measures ANOVA followed by Bonferroni's post hoc test (multiple groups at different time points). Pearson's correlation coefficient was chosen for evaluating the relationship between two variables. A *p* < 0.05 was described as statistically significant.

## 3. Results

### 3.1. BMSC-EVs Deliver NEAT1 to Chondrocytes

**Figure 1:**
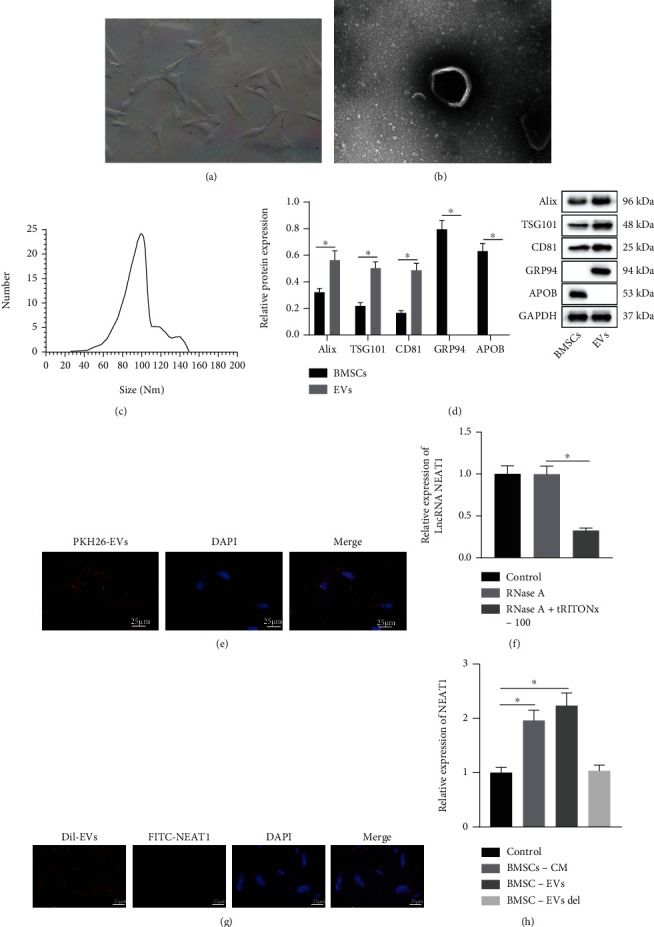
BMSCs-EVs transfer NEAT1 into chondrocytes. (a) Morphology of BMSCs at passage 3 observed under a light microscope. (b) Morphology of BMSC-EVs observed by a TEM. (c) Diameter of BMSC-EVs detected by NTA. (d) EV marker proteins Alix, TSG101, CD81, cellular endoplasmic reticulum protein GRP94, and plasma lipoprotein APOB in the BMSC-EVs detected by Western blot analysis. (e) Uptake of PKH26-labeled EVs by chondrocytes observed under a fluorescent microscope. EVs labeled by PKH67 (red); nuclei stained by DAPI (blue) (400×; scale bar = 25 *μ*m). (f) NEAT1 expression in EVs after treatment of RNase A or combined with Triton X-100 determined by RT-qPCR. (g) Uptake of BMSC-EVs containing FITC-NEAT1 by chondrocytes observed under a fluorescent microscope (400×; scale bar = 25 *μ*m). (h) NEAT1 expression in chondrocytes after coculture with BMSC-EVs detected by RT-qPCR. ∗*p* < 0.05. BMSC-CM is the conditioned medium for BMSCs, and BMSC-EVs del is the EV-free medium. Measurement data were expressed as mean ± standard deviation. Data between groups were compared using independent sample *t* test, while data among groups were compared by one-way ANOVA followed by Tukey's post hoc test. The cell experiment was run in triplicate independently.

BMSCs were isolated from human bone marrow and then purified. Under a light microscope, the isolated BMSCs were long fusiform or spindle-shaped with colony growth, and cells were aligned like a whirlpool when dense (Figure 1(a)). Flow cytometry showed that the surface markers of BMSCs at passage 3, CD73, CD90, CD44, and CD105 had more than 95% of positive rate, while the positive rate of CD34, CD45, and CD14 was below 2% (Supplementary Figure 1). The results indicated that the isolated BMSCs were in high purity, which were suitable for subsequent assays. Furthermore, the vesicles isolated from BMSCs were in double-membrane, with a diameter of 30-120 nm (Figures 1(b) and 1(c)). Meanwhile, Western blot analysis results manifested that ALIX, TSG101, and CD81 were detected in EVs, while non-EV marker (GRP94) and plasma lipoprotein (APOB) were negative (Figure 1(d)). These data showed the successful isolation of EVs from BMSCs.

To verify whether BMSC-EVs could be internalized by chondrocytes, EVs labeled by PKH26 were cocultured with chondrocytes *in vitro* for 24 h. Under the fluorescent microscope, more than 70% chondrocytes were in PKH26-positive (Figure 1(e)), which showed that EVs were effectively transferred into chondrocytes. Through the online tool (http://www.exorbase.org/), NEAT1 existed in circulating EVs and multiple kinds of cells. Furthermore, RNase protection experiment results revealed that NEAT1 content did not vary in culture medium, but was lowered after simultaneous treatment by RNase A and Triton X-100. Therefore, NEAT1 was wrapped by membranes, instead of being directly released (Figure 1(f)). The transferability of NEAT1 by BMSC-EVs was further explored. FITC-NEAT1 (green) was transferred into BMSCs to extract EVs which were then added with Dil tag (red) and cocultured with chondrocytes for 48 h. Fluorescence microscopic results showed that chondrocytes internalized BMSC-EVs containing FITC-NEAT1 (Figure 1(g)). Additionally, NEAT1 showed higher expression in the BMSCs + cultured medium group and the BMSCs + EVs group, but did not differ in the BMSCs + EVs del group compared with that in the control group (Figure 1(h)). Thus, NEAT1 could be transferred into chondrocytes through BMSC-EVs.

### 3.2. NEAT1 Delivered by BMSC-EVs Promotes the Proliferation and Autophagy of Chondrocytes while Suppressing Their Apoptosis

**Figure 2:**
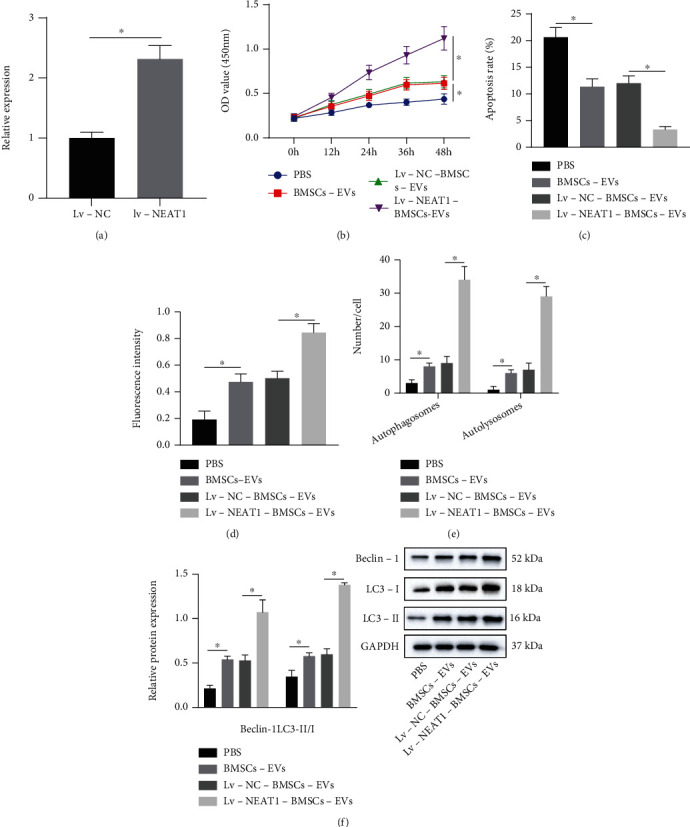
NEAT1 delivered by BMSC-EVs facilitates chondrocyte proliferation and autophagy but impairs their apoptosis. (a) NEAT1 expression in BMSC-EVs upon treatment with Lv-NEAT1 determined by RT-qPCR. (b) Proliferation of chondrocytes co-cultured with BMSC-EVs or Lv-NEAT1-BMSC-EVs detected by CCK-8. (c) Apoptosis of chondrocytes cocultured with BMSC-EVs or Lv-NEAT1-BMSC-EVs measured by flow cytometry. (d) Autophagy of chondrocytes co-cultured with BMSC-EVs or Lv-NEAT1-BMSC-EVs measured by MDC staining. (e) Formation of autophagosomes and autolysosome in chondrocytes cocultured with BMSC-EVs or Lv-NEAT1-BMSC-EVs analyzed by mRFP-GFP-LC3 double fluorescence assay. (f) Beclin-1 protein expression, as well as the ratio of LC3-II/I in chondrocytes cocultured with BMSC-EVs or Lv-NEAT1-BMSC-EVs analyzed by Western blot analysis. ∗*p* < 0.05. Measurement data were expressed as mean ± standard deviation. Data between groups were compared using independent sample *t* test, while data among groups were compared by one-way ANOVA followed by Tukey's post hoc test. The cell experiment was run in triplicate independently.

Next, the effect of NEAT1 delivered by BMSC-EVs on chondrocyte function was investigated. It was exhibited that NEAT1 expression was increased in BMSC-EVs following treatment with Lv-NEAT1 (Figure 2(a)). Besides, the proliferation of chondrocytes cocultured with BMSC-EVs was promoted while cell apoptosis was reduced. However, the proliferation of chondrocytes after treatment with Lv-NEAT1-BMSC-EVs was further enhanced, and cell apoptosis exhibited a more pronounced decline (Figures 2(b) and 2(c)). Besides, coculture with BMSC-EVs enhanced fluorescence intensity in chondrocytes, which was more obvious in the presence of Lv-NEAT1-BMSC-EVs (Figure 2(d)). The results of the mRFP-GFP-LC3 double fluorescence assay noted that the number of autophagosomes and autolysosome was increased in the chondrocytes cocultured with BMSC-EVs, with a more prominent increase noted following Lv-NEAT1-BMSC-EVs (Figure 2(e)). Moreover, Beclin-1 protein level and the ratio of LC3-II/I were increased in the chondrocytes co-cultured with BMSC-EVs, which was more evident following the treatment with Lv-NEAT1-BMSC-EVs (Figure 2(f)). Taken together, BMSC-EVs carrying NEAT1 accelerated the proliferation and autophagy of chondrocytes while arresting their apoptosis.

### 3.3. NEAT1 Binds to miR-122-5p to Induce the Proliferation and Autophagy of Chondrocytes while Arresting Their Apoptosis

**Figure 3:**
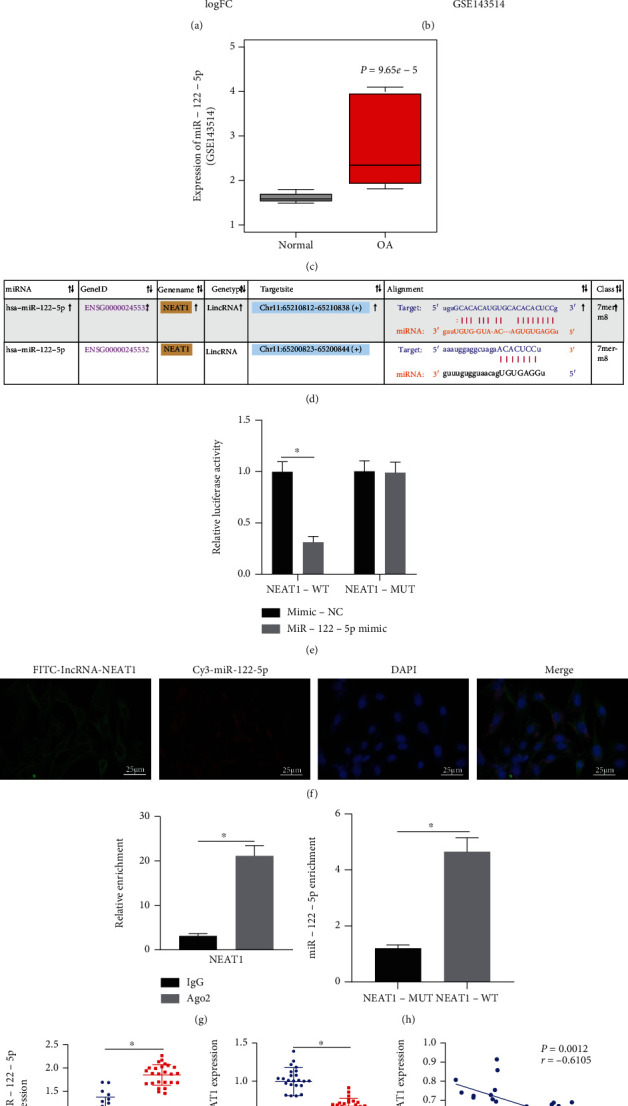
NEAT1 binds to miR-122-5p and downregulates its expression. (a) Differential analysis of miRNA expression in OA-related GSE143514 microarray. (b) Venn diagram of the predicted downstream miRNAs of NEAT1 by the starBase database and the upregulated miRNAs in OA samples in the OA-related GSE143514 microarray. (c) Expression of miR-122-5p in OA samples in the OA-related GSE143514 microarray. (d) Binding sites between NEAT1 and miR-122-5p. (e) Luciferase activity of NEAT1-WT and NEAT1-MUT detected by dual-luciferase reporter assay. (f) Colocalization of Cy3-miR-122-5p (red) and FITC-NEAT1 (green) determined by FISH (scale bar = 25 *μ*m). Nuclei were stained in blue by DAPI. (g) Interaction between NEAT1 and miR-122-5p detected by RIP. (h) Interaction between NEAT1 and miR-122-5p determined by RNA pull-down. (i) NEAT1 and miR-122-5p expression in cartilage tissues of OA patients (*n* = 25) and normal tissues (*n* = 25) (left) determined by RT-qPCR as well as the correlation analysis of NEAT1 expression and miR-122-5p expression in cartilage tissues of OA patients (*n* = 25) (right) by Pearson's correlation coefficient. ∗*p* < 0.05. Measurement data were expressed as mean ± standard deviation. Data between groups were compared using independent sample *t* test. Correlation was analyzed by Pearson's correlation analysis. The cell experiment was run in triplicate independently.

The mechanism of NEAT1 carried by BMSC-EVs in OA was in-depth studied. starBase database predicted the downstream miRNAs of NEAT1 (Supplementary Figure 2). OA-related miRNA microarray data GSE143514 was retrieved through GEO database, differential analysis of which revealed 8 upregulated and 48 downregulated miRNAs in OA samples (Figure 3(a)). Following intersection analysis of the prediction results by the starBase database with the upregulated miRNAs in the GSE143514 microarray, only miR-122-5p, miR-205-5p, and miR-138-5p were found at the intersection (Figure 3(b)). Of them, miR-122-5p was upregulated in OA samples in the GSE143514 microarray (Figure 3(c)). In addition, there existed binding site between NEAT1 and miR-122-5p (Figure 3(d)). The above results indicated the critical role of NEAT1 in OA by regulating miR-122-5p.

Luciferase assay data displayed that overexpression of miR-122-5p suppressed the luciferase activity of NEAT1-WT, but did not affect that of NEAT1-MUT (Figure 3(e)). Also, FISH assay results described the colocalization of NEAT1 and miR-122-5p in cells (Figure 3(f)). In addition, RIP assay results demonstrated that anti-Ago2 antibody could significantly enrich NEAT1 (Figure 3(g)), suggesting that NEAT1 might exist in the miR-122-5p RISC complex. RNA pull-down results showed that the expression of miR-122-5p in the NEAT1-WT pull-down pellets was diminished (Figure 3(h)). Meanwhile, NEAT1 expression was diminished while miR-122-5p was highly expressed in cartilage tissues of OA patients. Pearson's correlation coefficient suggested an adverse correlation of NEAT1 expression with miR-122-5p in cartilage tissues of OA patients (Figure 3(i)).

**Figure 4:**
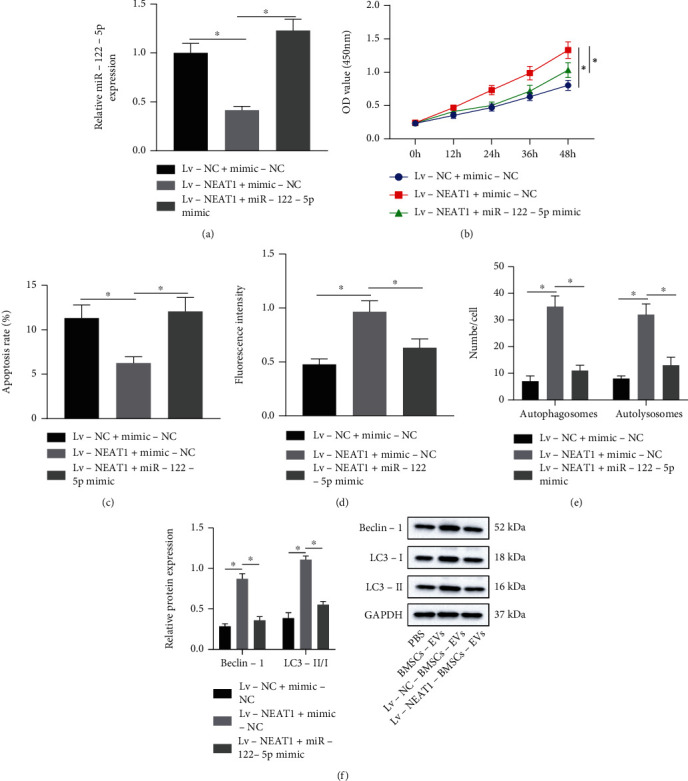
NEAT1 promotes proliferation and autophagy of chondrocytes but impairs their apoptosis by binding to miR-122-5p. (a) miR-122-5p expression in chondrocytes treated with Lv-NEAT1 or combined with miR-122-5p mimic detected by RT-qPCR. (b) Proliferation of chondrocytes treated with Lv-NEAT1 or combined with miR-122-5p mimic detected by CCK-8. (c) Apoptosis of chondrocytes treated with Lv-NEAT1 or combined with miR-122-5p mimic measured by flow cytometry. (d) Autophagy of chondrocytes treated with Lv-NEAT1 or combined with miR-122-5p mimic measured by MDC staining. (e) Formation of autophagosomes and autolysosome in chondrocytes treated with Lv-NEAT1 or combined with miR-122-5p mimic analyzed by mRFP-GFP-LC3 double fluorescence assay. (f) Beclin-1 protein expression, as well as the ratio of LC3-II/I in chondrocytes treated with Lv-NEAT1 or combined with miR-122-5p mimic analyzed by Western blot analysis. ∗*p* < 0.05. Measurement data were expressed as mean ± standard deviation. Data among groups were compared by one-way ANOVA followed by Tukey's post hoc test. Data at different time points were compared using repeated-measures ANOVA followed by Bonferroni's post hoc test. The cell experiment was run in triplicate independently.

RT-qPCR results documented that miR-122-5p expression was reduced in chondrocytes overexpressing NEAT1, which was neutralized by treatment with Lv − NEAT1 + miR − 122 − 5p mimic (Figure 4(a)). This indicated that NEAT1 reduced miR-122-5p expression by binding to miR-122-5p. Moreover, NEAT1 overexpression caused elevated chondrocyte viability and reduced cell apoptosis, which was counteracted by miR-122-5p mimic (Figures 4(b) and 4(c)). Also, the results of MDC staining and mRFP-GFP-LC3 double fluorescence assay described that fluorescence intensity was strengthened, and the number of autophagosomes and autolysosomes was increased in chondrocytes overexpressing NEAT1, but these results were normalized by miR-122-5p mimic (Figures 4(d) and 4(e)). Results of Western blot analysis further exhibited augmented Beclin-1 protein level and ratio of LC3-II/I in chondrocytes overexpressing NEAT1, which was annulled by miR-122-5p mimic (Figure 4(f)).

### 3.4. NEAT1 Upregulates Sesn2 and Activates Nrf2 Pathway by Binding to miR-122-5p

**Figure 5:**
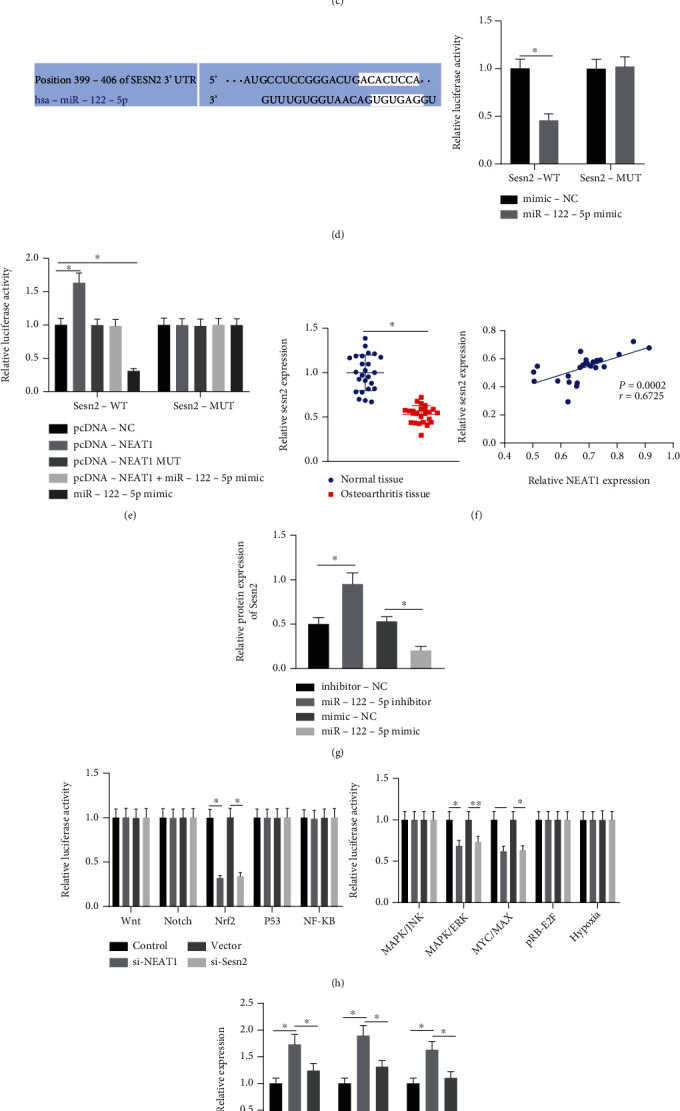
NEAT1 binds to miR-122-5p, thus upregulating Sesn2 expression and activating the Nrf2 pathway. (a) Differential expression of mRNAs in OA samples in OA-related GSE16464 microarray. (b) Venn diagram of the predicted downstream mRNAs of miR-122-5p by the starBase, TargetScan, and miRDB databases and the downregulated mRNAs in OA samples in the OA-related GSE16464 microarray. (c) Expression of Sesn2 in OA samples in the GSE16464 microarray. (d) Binding sites between Sesn2 and miR-122-5p predicted by TargetScan and their binding in HEK-293T cells confirmed by dual-luciferase reporter assay. (e) Interaction among NEAT1, miR-122-5p, and Sesn2 detected by dual-luciferase reporter assay. (f) Sesn2 expression in cartilage tissues of OA patients (*n* = 25) and normal tissues (*n* = 25) detected by RT-qPCR (left) as well as the correlation analysis of NEAT1 expression and Sesn2 expression in cartilage tissues of OA patients (*n* = 25) by Pearson's correlation coefficient (right). (g) Sesn2 protein expression in chondrocytes transfected with miR-122-5p mimic or miR-122-5p inhibitor determined by Western blot analysis. (h) Protein expression of the Nrf2 pathway-related factors in chondrocytes transfected with si-Nrf2 or si-NEAT1 determined by Western blot analysis. (i) mRNA expression of Sesn2, Srx1, and Trx1 in chondrocytes treated with oe-NEAT1 or combined with miR-122-5p mimic detected by RT-qPCR. (j) Protein expression of Sesn2, Nrf2, Srx1, and Trx1 in chondrocytes treated with oe-NEAT1 or combined with miR-122-5p mimic detected by Western blot analysis. ∗*p* < 0.05. Measurement data were expressed as mean ± standard deviation. Data between groups were compared using independent sample *t* test. Correlation was analyzed by Pearson's correlation analysis. Data among groups were compared by one-way ANOVA followed by Tukey's post hoc test. The cell experiment was run in triplicate independently.

Aiming to dissect out the downstream mechanism of NEAT1/miR-122-5p in OA, we predicted downstream target of miR-122-5p through starBase, TargetScan, and miRDB databases (Supplementary Figure 3). Differentially expressed mRNAs in OA samples in OA-related microarray GSE16464 were analyzed, with 258 upregulated mRNAs and 185 downregulated mRNAs obtained (Figure 5(a)). The downregulated mRNAs in the GSE16464 microarray were intersected with the above prediction results, with only Sesn2 found at the intersection (Figure 5(b)). Meanwhile, Sesn2 expression was obviously downregulated in OA samples in the GSE16464 microarray (Figure 5(c)). TargetScan predicted the binding sites between miR-122-5p and Sesn2 which were validated utilizing luciferase assay that the luciferase activity of Sesn2-WT was decreased in HEK-293T cells transfected with miR-122-5p mimic, but that of Sesn2-MUT was not altered (Figure 5(d)). Also, transfection with pcDNA-NEAT1 enhanced the luciferase activity of Sesn2-WT, which was not affected by pcDNA-NEAT1-MUT. Higher luciferase activity of Sesn2-WT was found in response to pcDNA − NEAT1 + miR − 122 − 5p mimic than miR-122-5p mimic alone but the luciferase activity of Sesn2-MUT did not differ upon each treatment (Figure 5(e)). Through RT-qPCR, Sesn2 expression was higher in cartilage tissues of OA patients. Additionally, Sesn2 shared positive correlated with NEAT1 expression in cartilage tissues of OA patients (Figure 5(f)). Sesn2 protein expression was reduced in chondrocytes after gain-of-function of miR-122-5p, but it was enhanced after loss-of-function of miR-122-5p (Figure 5(g)). This indicated that miR-122-5p could target Sesn2 and inhibit the expression of Sesn2. Moreover, after silencing of NEAT1 or Sesn2, activity of the Nrf2 pathway was reduced (Figure 5(h)). Further, we found that Sesn2, Nrf2, Srx1, and Trx1 expressions were elevated in NEAT1-overexpressing chondrocytes, which was nullified by miR-122-5p mimic (Figures 5(i) and 5(j)).

### 3.5. NEAT1 Delivered by BMSC-EVs Boosts Chondrocyte Proliferation and Autophagy while Impeding Cell Apoptosis via the miR-122-5p/Sesn2 Axis

**Figure 6:**
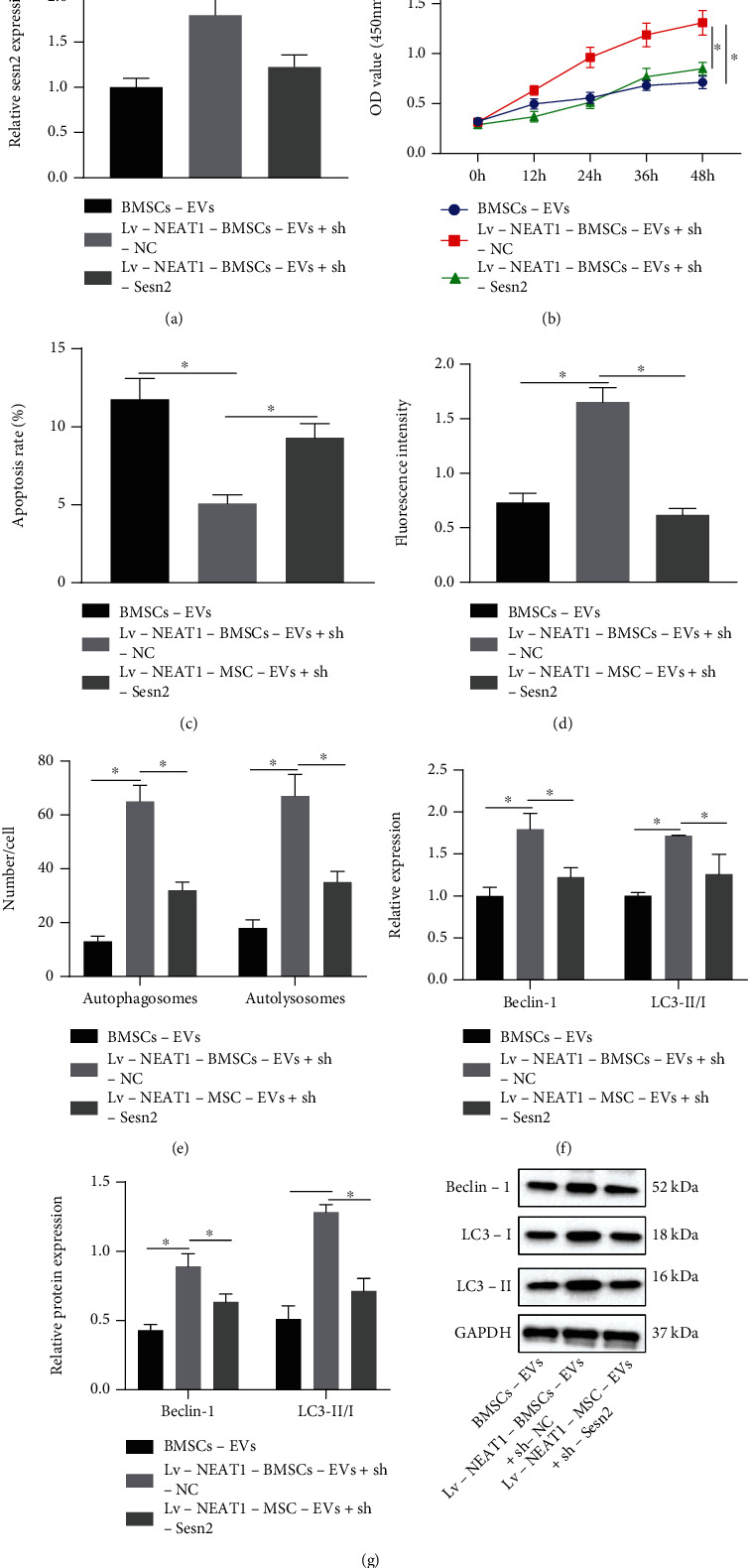
NEAT1 delivered by BMSC-EVs induces proliferation and autophagy of chondrocytes but represses their apoptosis via the miR-122-5p/Sesn2 axis. (a) Sesn2 mRNA expression in chondrocytes treated with Lv-NEAT1-BMSC-EVs or combined with sh-Sesn2 detected by RT-qPCR. (b) Proliferation of chondrocytes treated with Lv-NEAT1-BMSC-EVs or combined with sh-Sesn2 detected by CCK-8. (c) Apoptosis of chondrocytes treated with Lv-NEAT1-BMSC-EVs or combined with sh-Sesn2 measured by flow cytometry. (d) Autophagy of chondrocytes treated with Lv-NEAT1-BMSC-EVs or combined with sh-Sesn2 measured by MDC staining. (e) Formation of autophagosomes and autolysosome in chondrocytes treated with Lv-NEAT1-BMSC-EVs or combined with sh-Sesn2 analyzed by mRFP-GFP-LC3 double fluorescence assay. (f) Beclin-1 and LC3-II/I mRNA expression in chondrocytes treated with Lv-NEAT1-BMSC-EVs or combined with sh-Sesn2 analyzed by RT-qPCR. (g) Beclin-1 protein expression and the ratio of LC3-II/I in chondrocytes treated with Lv-NEAT1-BMSC-EVs or combined with sh-Sesn2 analyzed by Western blot analysis. ∗*p* < 0.05. Measurement data were expressed as mean ± standard deviation. Data among groups were compared by one-way ANOVA followed by Tukey's post hoc test. Data at different time points were compared using repeated-measures ANOVA followed by Bonferroni's post hoc test. The cell experiment was run in triplicate independently.

The effects of NEAT1 delivered by BMSC-EVs regulating the miR-122-5p/Sesn2 axis on the biological functions of chondrocytes were further explored by treating chondrocytes with BMSC-EVs, Lv − NEAT1 − BMSC − EVs + sh − NC, or Lv − NEAT1 − BMSC − EVs + sh − Sesn2. It was described that Sesn2 mRNA expression was augmented in chondrocytes treated with Lv-NEAT1-BMSC-EVs, which was reduced by further sh-Sesn2 treatment (Figure 6(a)). Functional assays indicated that chondrocyte viability was increased, and apoptosis was weakened in response to treatment with Lv-NEAT1-BMSC-EVs, which was neutralized by further sh-Sesn2 (Figures 6(b) and 6(c)). Moreover, fluorescence intensity was strengthened, the number of autophagosomes and autolysosome was increased, and Beclin-1 protein level and the ratio of LC3-II/I were enhanced in chondrocytes treated with Lv-NEAT1-BMSC-EVs, indicatives of augmented cell autophagy, which was neutralized by further sh-Sesn2 (Figures 6(d)–6(g)). To sum up, NEAT1 carried by BMSC-EVs could downregulate miR-122-5p expression to increase Sesn2 expression, promoting the proliferation and autophagy of chondrocytes but inhibit their apoptosis.

### 3.6. NEAT1 Delivered by BMSC-EVs Suppresses OA Progression by Regulating the miR-122-5p/Sesn2/Nrf2 Axis In Vivo

**Figure 7:**
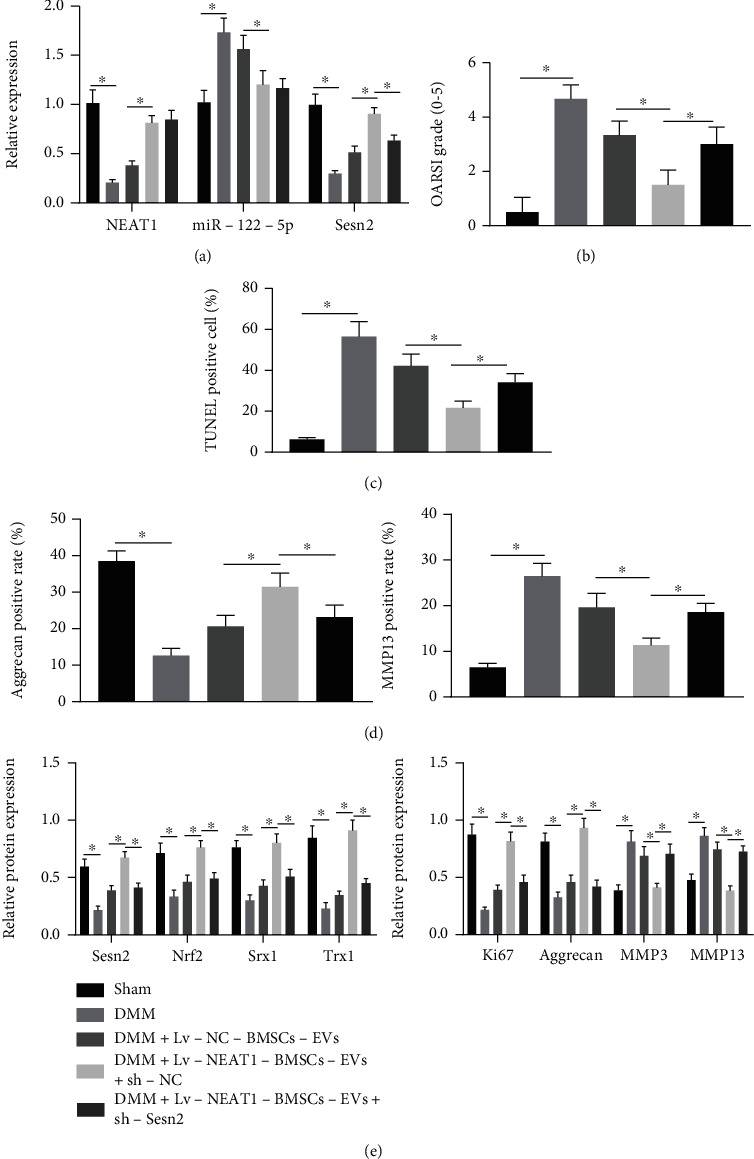
NEAT1 delivered by BMSC-EVs ameliorates OA via the miR-122-5p/Sesn2/Nrf2 axis in vivo (*n* = 6 for mice in each group). (a) NEAT1 and Sesn2 mRNA expression and miR-122-5p expression in cartilage tissues of OA mice treated with Lv-NEAT1-BMSC-EVs or combined with sh-Sesn2 detected by RT-qPCR. (b) OARSI scoring of OA mice treated with Lv-NEAT1-BMSC-EVs or combined with sh-Sesn2. (c) Cell apoptosis in cartilage tissues of OA mice treated with Lv-NEAT1-BMSC-EVs or combined with sh-Sesn2 determined by TUNEL staining. (d) MMP13 and Aggrecan positive expression in cartilage tissues of OA mice treated with Lv-NEAT1-BMSC-EVs or combined with sh-Sesn2 measured by immunohistochemistry. (e) Sesn2, Nrf2, Srx1, Trx1, Ki67, MMP3, MMP13, and Aggrecan protein expression in cartilage tissues of OA mice treated with Lv-NEAT1-BMSC-EVs or combined with sh-Sesn2 analyzed by Western blot analysis. ∗*p* < 0.05. Measurement data were expressed as mean ± standard deviation. Data among groups were compared by one-way ANOVA followed by Tukey's post hoc test.

To pinpoint the impacts of NEAT1 regulating the miR-122-5p/Sesn2/Nrf2 axis on OA *in vivo*, an OA model was first established in mice by DMM and then treated with Lv-NEAT1-BMSC-EVs and sh-Sesn2. RT-qPCR indicated a decline in the NEAT1 and Sesn2 expression but an elevation in miR-122-5p expression in cartilage tissues of DMM mice. However, NEAT1 and Sesn2 expressions were elevated in cartilage tissues of DMM mice treated with Lv-NEAT1-BMSC-EVs, while miR-122-5p was downregulated. Furthermore, Sesn2 expression was reduced in cartilage tissues of DMM mice by sh-Sesn2 in the presence of Lv-NEAT1-BMSC-EVs, whereas NEAT1 and Sesn2 expression did not significantly change (Figure 7(a)). OARSI scoring results showed high score in DMM mice. However, OARSI score was lowered in DMM mice by Lv-NEAT1-BMSC-EVs, which was restored by further silencing Sesn2 (Figure 7(b)). Cell apoptosis was enhanced in cartilage tissues of DMM mice. Furthermore, DMM mice treated with Lv-NEAT1-BMSC-EVs displayed reduced cell apoptosis, which was rescued by further Sesn2 silencing (Figure 7(c)). Immunohistochemistry results showed that the positive expression of Aggrecan was decreased and that of MMP13 was augmented in cartilage tissues of DMM mice. However, the positive expression of Aggrecan was elevated and that of MMP13 was diminished upon treatment with Lv-NEAT1-BMSCs-EVs, the effect of which was abolished by further sh-Sesn2 (Figure 7(d)). Western blot analysis further manifested that Sesn2, Nrf2, Srx1, Trx1, Ki67, and Aggrecan protein expressions were reduced but MMP3 and MMP13 protein expressions were upregulated in cartilage tissues of DMM mice. In cartilage tissues of DMM mice treated with Lv-NEAT1-BMSC-EVs, Sesn2, Nrf2, Srx1, Trx1, Ki67, and Aggrecan protein expression were upregulated but MMP3 and MMP13 protein expressions were downregulated, while in the presence of Lv − NEAT1 − BMSC − EVs + sh − Sesn2, opposite results were noted (Figure 7(e)). These results indicated that NEAT1 delivered by BMSC-EVs suppressed OA by regulating the miR-122-5p/Sesn2/Nrf2 axis *in vivo.*

## 4. Discussions

Among the currently available OA treatments, intra-articular delivery of biologics against molecules or cells show potentials, including biological therapies, cell therapies, and growth factor therapy [27]. The therapeutic effects of EVs from BMSCs on OA have been reported [28]. However, a comprehensive understanding regarding the mechanism of EVs derived from BMSCs in OA has not been determined. Therefore, a mouse model and chondrocytes were treated with BMSC-EVs in our study to explore how NEAT1 from BMSCs-EVs functions in OA progression and whether NEAT1 affects the cell growth by regulation of potential related molecules. Consequently, our findings denoted that BMSC-EVs carrying NEAT1 bound to miR-122-5p, leading to activation of the Sesn2/Nrf2 axis, which alleviated OA both *in vitro* and *in vivo.*

Our study began with successful isolation of EVs from BMSCs, as evidenced by the positive expression patterns of ALIX, TSG101, and CD81. An existing study has determined ALIX, TSG101, and CD81 as characteristic markers for EV [29]. In subsequent analysis, our findings revealed that NEAT1 was encapsulated in the membrane of EVs. Previously, a study detected the presence of NEAT1 in large EVs [18]. Furthermore, our results validated the ability of NEAT1 to radically accelerate chondrocyte proliferation and restrain apoptosis. Our results were in consistency with an existing study highlighting the downregulation of NEAT1 in OA tissues and the capacity of NEAT1 expression silencing to aggravate OA [30]. In line with an existing study, NEAT1 facilitates the proliferation of chondrocytes, but suppresses cell apoptosis in OA [31]. Furthermore, our results highlighted that NEAT1 promoted chondrocyte autophagy. Curtailed chondrocyte autophagy shares closely related to the progression of OA [32]. Therefore, NEAT1-based promotion of chondrocyte autophagy was indicative of OA amelioration. The aforementioned literature supported our results eliciting that BMSCs-EVs delivering NEAT1 relieved OA by inducing the proliferation and autophagy of chondrocytes.

In regard to the potential therapeutic effects of NEAT1 on OA, our study focused on investigating the downstream molecular mechanism. Bioinformatics study and luciferase assay revealed that NEAT1 bound to miR-122-5p. Coincidentally, miR-122 has been identified as a target of NEAT1, where a lowered NEAT1 expression was resultant of upregulated miR-122 [20], which consistent with our results eliciting that NEAT1 was negatively correlated with miR-122-5p. An existing study identified an increase in the miR-122-5p expression in knee OA [21]. Our findings determined Sesn2 as a target gene of miR-122-5p. Low Sesn2 is presented in OA tissues [33]. Additionally, lowered Sesn mRNA level is seen in human OA cartilage, where Sesn can support the survival of chondrocytes under stress to ameliorate OA by facilitating mTOR-dependent autophagy [22]. Moreover, Sesn2 overexpression could elevate the total nuclear level of Nrf2 as well as the downstream protein Nrf2, while knockdown of Sesn2 reduces the level of Nrf2, that is, overexpression of Sesn2 activates the Nrf2 pathway [34]. Consistently, prior research manifested that activation of Nrf2 pathway could attenuate extracellular matrix degradation in chondrocytes and murine OA [35].

## 5. Conclusions

**Figure 8:**
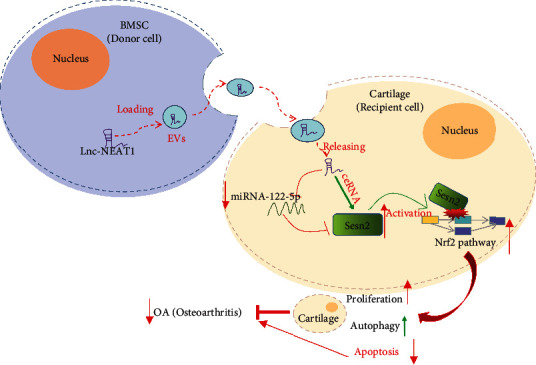
Schematic diagram of the mechanism by which NEAT1 affects OA progression. BMSC-EVs can deliver NEAT1 into chondrocytes where NEAT1 competitively binds to miR-122-5p and upregulates the expression of the miR-122-5p target Sesn2, thereby activating the Nrf2 pathway, promoting chondrocyte proliferation and autophagy while inhibiting their apoptosis, ultimately preventing the progression of OA.

To conclude, our results elicited that targeting NEAT1 could downregulate miR-122-5p expression so as to elevate the Sesn2 expression and chondrocyte proliferation could serve as a promising strategy for OA treatment (Figure 8). Future studies are warranted to accelerate the development of BMSCs-EVs-based target therapies. In addition, published literature has confirmed that artificial exosomes can carry specific molecules and deliver them to target cells [36]. Therefore, the delivery of NEAT1 or miR-122-5p inhibitor into chondrocytes through artificial EVs will be an ideal treatment option for OA. However, how to make artificial EVs target the target cells will pose a great challenge.

## Data Availability

The datasets generated/analyzed during the current study are available from the corresponding author upon request.

## Abbreviations

OA: Osteoarthritis
EVs: Extracellular vesicles
BMSCs: Bone marrow mesenchymal stem cells
lncRNAs: Long noncoding RNAs
NEAT: Nuclear paraspeckle assembly transcript
Sesn: Sestrins
PBS: Phosphate buffer saline
Lv: Lentivirus
GFP: Green fluorescent protein
MDC: Monodansylcadaverine
DMEM: Dulbecco's modified Eagle's medium
POD: Peroxidase
miRNAs or miRs: MicroRNAs
GEO: Gene Expression Omnibus
oe: Overexpression
siRNA: Small interfering RNA
NC: Negative control
sh: Short hairpin
DAB: 3,3′-Diaminobenzidine tetrahydrochloride
3′-UTR: 3′-Untranslated region
BSA: Bovine serum albumin
TUNEL: Terminal deoxynucleotidyl transferase-mediated dUTP-biotin nick end labeling
PI: Propidium iodide
FITC: Fluorescein isothiocyanate
FBS: Fetal bovine serum
cDNA: Complementary DNA
RT-qPCR: Reverse transcription-quantitative polymerase chain reaction
MMP: Matrix metalloproteinase
GAPDH: Glyceraldehyde-3-phosphate dehydrogenase
RIPA: Radio-immunoprecipitation assay
BCA: Bicinchoninic acid
SDS-PAGE: Sodium dodecyl sulfate-polyacrylamide gel electrophoresis
PVDF: Polyvinylidene fluoride
HRP: Horseradish peroxidase
DAPI: 4′,6-Diamidino-2-phenylindole
FISH: Fluorescent in situ hybridization
WT: Wild-type
MUT: Mutant
RIP: RNA binding protein immunoprecipitation
Ago2: Argonaute2
IgG: Immunoglobulin G
ANOVA: Analysis of variance.

## Supplementary Materials

Supplementary 1 Supplementary Figure 1: surface antigens of BMSCs, CD73, CD90, CD44, and CD105, as well as non-BMSC surface antigens CD34, CD45, and CD14 determined by flow cytometry. Supplementary Figure 2: regulatory miRNAs of NEAT1 downstream predicted by StarBase database. Supplementary Figure 3: downstream target genes of miR-122-5p predicted by StarBase, TargetScan, and miRDB databases.

Supplementary 2 Supplementary Table 1: primer sequences for RT-qPCR. Supplementary Table 2: OARSI scoring system.
