# Supplementary material for: Bone Mesenchymal Stem Cell-Derived Extracellular Vesicles Containing Long Noncoding RNA NEAT1 Relieve Osteoarthritis
Source: Oxid Med Cell Longev. 2022 Apr 15;2022:5517648. doi: 10.1155/2022/5517648 (PMC9036164; doi:10.1155/2022/5517648)
Supplement: Supplementary 1 — Supplementary Figure 1: surface antigens of BMSCs, CD73, CD90, CD44, and CD105, as well as non-BMSC surface antigens CD34, CD45, and CD14 determined by flow cytometry. Supplementary Figure 2: regulatory miRNAs of NEAT1 downstream predicted by StarBase database. Supplementary Figure 3: downstream target genes of miR-122-5p predicted by StarBase, TargetScan, and miRDB databases. [file 5517648.f1.docx]

**
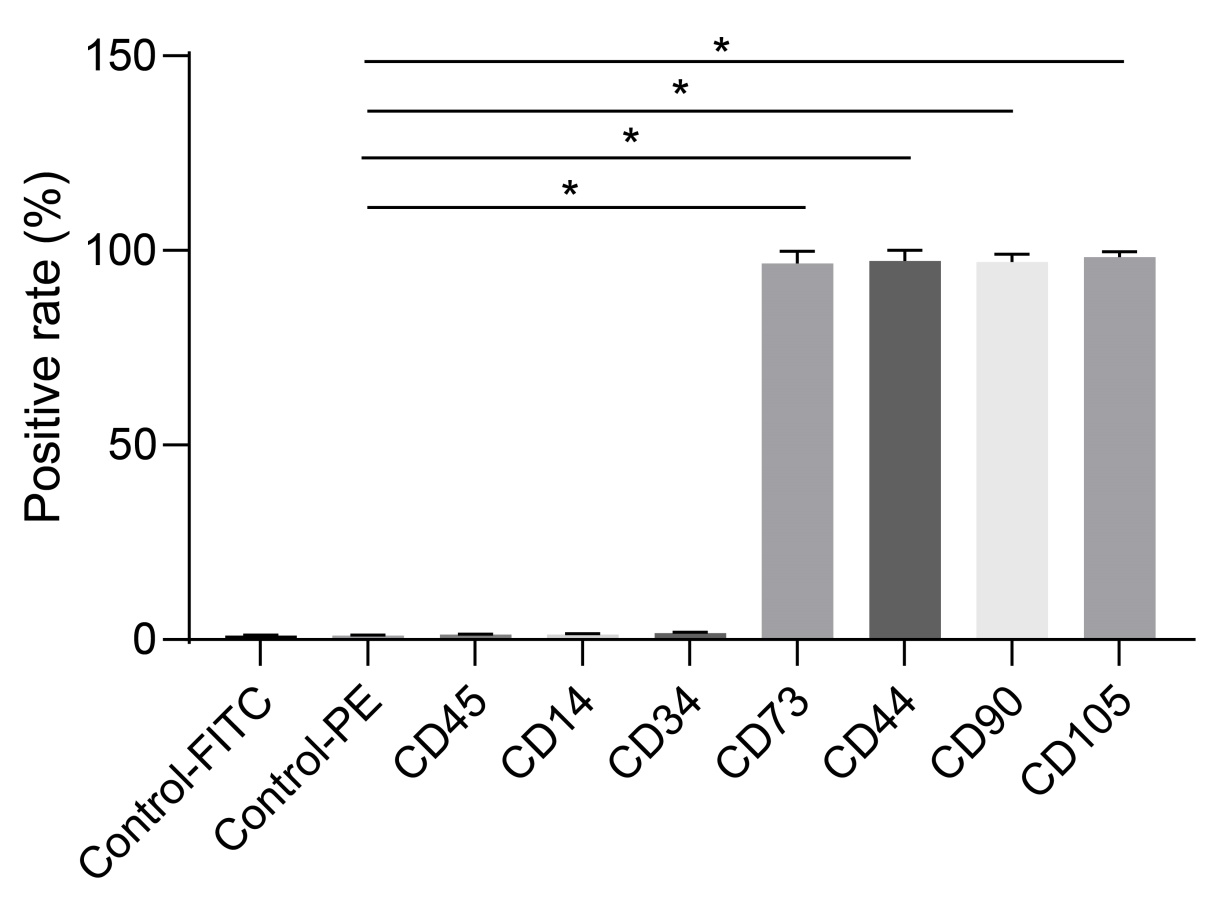
**

**SUPPLEMENTARY FIGURE 1** Surface antigens of BMSCs, CD73, CD90, CD44, and CD105, as well as non-BMSC surface antigens CD34, CD45, and CD14 determined by flow cytometry.

**
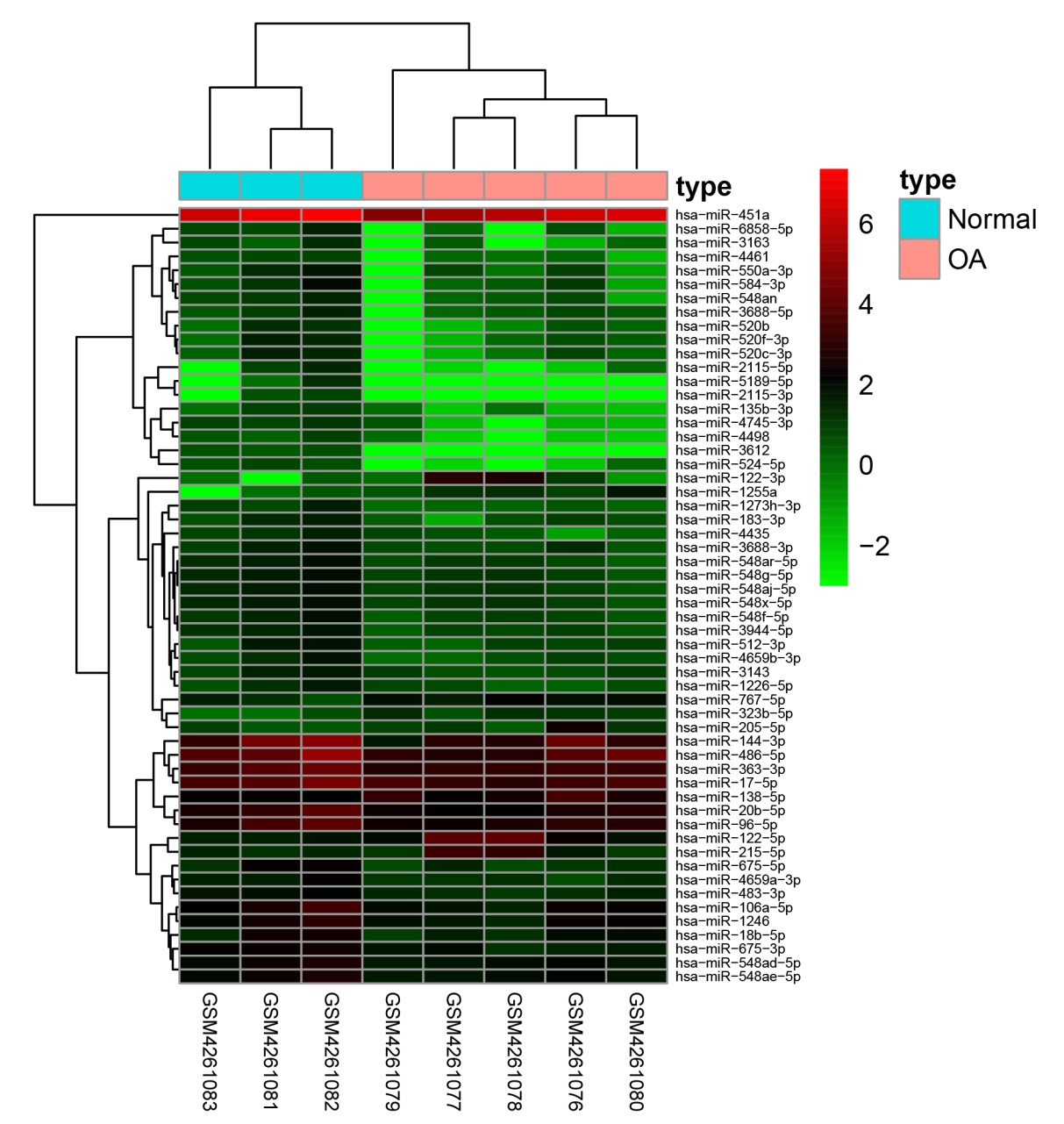
**

**SUPPLEMENTARY FIGURE 2** Regulatory miRNAs of NEAT1 downstream predicted by StarBase database.

**
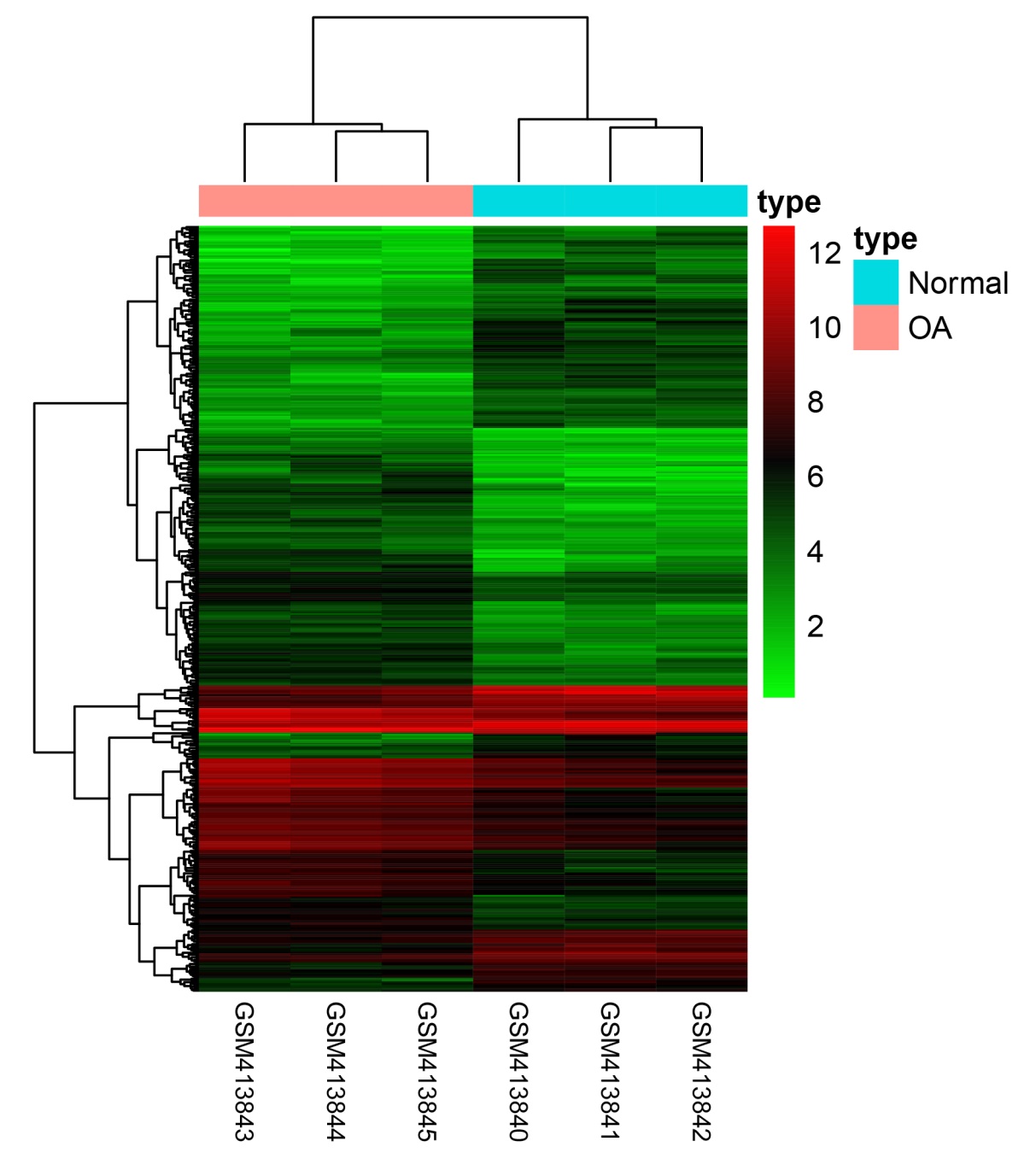
**

**SUPPLEMENTARY FIGURE 3** Downstream target genes of miR-122-5p predicted by StarBase, TargetScan, and miRDB databases.
