# Supplementary material for: Bone Mesenchymal Stem Cell-Derived Extracellular Vesicles Containing Long Noncoding RNA NEAT1 Relieve Osteoarthritis
Source: Oxid Med Cell Longev. 2022 Apr 15;2022:5517648. doi: 10.1155/2022/5517648 (PMC9036164; doi:10.1155/2022/5517648)
Supplement: Supplementary 2 — Supplementary Table 1: primer sequences for RT-qPCR. Supplementary Table 2: OARSI scoring system. [file 5517648.f2.docx]

**SUPPLEMENTARY TABLE 1** Primer sequences for RT-qPCR

| Targets | Primer sequences (5'-3') |
| --- | --- |
| miR-122-5p | F: TGGAGTGTGACAATGGTGTTTG |
|  | R: Universal reverse primer |
| NEAT1 | F: CCACAACGCAGATTGATGCC |
|  | R: GAAACGCACAAGAAGGCAGG |
| Sesn2 | F: AAGGACTACCTGCGGTTCG |
|  | R: CGCCCAGAGGACATCAGTG |
| Nrf2 | F: TCAGCGACGGAAAGAGTATGA |
|  | R: CCACTGGTTTCTGACTGGATGT |
| Srx1 | F: CAGGGAGGTGACTACTTCTACTC |
|  | R: CAGGTACACCCTTAGGTCTGA |
| Trx1 | F: GTGAAGCAGATCGAGAGCAAG |
|  | R: CGTGGCTGAGAAGTCAACTACTA |
| U6 | F: CTCGCTTCGGCAGCACA |
|  | R: Universal reverse primer |
| GAPDH | F: AGAAGGCTGGGGCTCATTTG |
|  | R: AGGGGCCATCCACAGTCTTC |

Notes: RT-qPCR, reverse transcription quantitative polymerase chain reaction; miR-122-5p, microRNA-122-5p; NEAT1, nuclear paraspeckle assembly transcript 1; Sesn2, sestrin 2; Nrf2, nuclear factor erythroid 2-related factor 2; Srx1, sulphiredoxin; GAPDH, glyceraldehyde 3-phosphate dehydrogenase; F, forward; R, reverse.

**SUPPLEMENTARY TABLE 2** OARSI scoring system

| Grade (Key Feature) | Subgrade (Optional) |
| --- | --- |
| Grade 0: surface intact | No subgrade |
| Grade 1: uneven but intact surface | 1.0 Cell intact |
|  | 1.5 Cell death |
| Grade 2: surface discontinuity | 2.0 Fibrillation through superficial zone |
|  | 2.5 Surface abrasion with matrix loss within superficial zone |
| Grade 3: vertical fissures | 3.0 Simple fissures |
|  | 3.5 Branched/complex fissures |
| Grade 4: erosion | 4.0 Superficial zone delamination |
|  | 4.5 Mid zone excavation |
| Grade 5: denudation | 5.0 Bone surface intact |
